# High-Throughput Screening to Identify Inhibitors of *Plasmodium falciparum* Importin α

**DOI:** 10.3390/cells11071201

**Published:** 2022-04-02

**Authors:** Sujata B. Walunj, Manisha M. Dias, Chhaminder Kaur, Kylie M. Wagstaff, Vishakha Dey, Caroline Hick, Swati Patankar, David A. Jans

**Affiliations:** 1 Nuclear Signaling Laboratory, Department of Biochemistry and Molecular Biology, Monash Biomedicine Discovery Institute, Monash University, Clayton, VIC 3800, Australia; sujata.walunj@monash.edu (S.B.W.); manisha.treeby@monash.edu (M.M.D.); kylie.wagstaff@monash.edu (K.M.W.); 2 Molecular Parasitology Laboratory, Department of Biosciences and Bioengineering, IIT Bombay, Powai, Mumbai 400076, India; chhaminder.kaur@iitb.ac.in (C.K.); vishakhadey@gmail.com (V.D.); patankar@iitb.ac.in (S.P.); 3 IITB-Monash Research Academy, IIT Bombay, Mumbai 400076, India; 4 Monash Institute of Pharmaceutical Sciences, Monash University, Parkville, VIC 3052, Australia; caroline.hick@monash.edu

**Keywords:** *Plasmodium falciparum*, malaria, *Toxoplasma gondii*, toxoplasmosis, importins, nuclear import inhibitors

## Abstract

The global burden of malaria and toxoplasmosis has been limited by the use of efficacious anti-parasitic agents, however, emerging resistance in *Plasmodium* species and *Toxoplasma gondii* threatens disease control worldwide, implying that new agents/therapeutic targets are urgently needed. Nuclear localization signal (NLS)-dependent transport into the nucleus, mediated by members of the importin (IMP) superfamily of nuclear transporters, has shown potential as a target for intervention to limit viral infection. Here, we show for the first time that IMPα from *P. falciparum* and *T. gondii* have promise as targets for small molecule inhibitors. We use high-throughput screening to identify agents able to inhibit *P. falciparum* IMPα binding to a *P. falciparum* NLS, identifying a number of compounds that inhibit binding in the µM-nM range, through direct binding to *P. falciparum* IMPα, as shown in thermostability assays. Of these, BAY 11-7085 is shown to be a specific inhibitor of *P. falciparum* IMPα-NLS recognition. Importantly, a number of the inhibitors limited growth by both *P. falciparum* and *T. gondii*. The results strengthen the hypothesis that apicomplexan IMPα proteins have potential as therapeutic targets to aid in identifying novel agents for two important, yet neglected, parasitic diseases.

## 1. Introduction

Organisms from the phylum apicomplexa, such as *Plasmodium* spp., *Toxoplasma gondii*, *Cyclospora* spp., and *Cryptosporidium* spp., can cause severe disease in humans [1,2]. The World Health Organization (WHO) estimates close to 240 million malaria cases caused by *Plasmodium* spp. in 2020 [3], with >600,000 deaths, predominantly of children under the age of five. *T. gondii* chronically infects about one-third of the human population worldwide with the majority of infections being asymptomatic, however, infection of immunocompromised individuals (such as those suffering from autoimmune diseases syndrome—AIDS) can result in severe toxoplasmosis, whilst in the case of pregnant women, hydrocephaly/microcephaly can occur in new-born babies [4,5,6]. Emerging drug resistance to the current frontline chemotherapy is a major threat to the control of malaria and toxoplasmosis [6,7,8], with an accompanying urgent need for new drugs with novel mechanisms of action to control these diseases.

Signal-dependent transport into and out of the eukaryotic cell nucleus, mediated by members of the importin (IMP) superfamily of proteins, is central to processes such as cell differentiation, transformation, development, and infection and immunity [9,10,11,12,13]. In a classical nuclear import, IMPα recruits IMPβ1 through its IMPβ-binding domain (IBB) and then binds to the nuclear localization signal (NLS) of cargo proteins to be subsequently imported into the nucleus [10,11,14]. In the absence of IMPβ1, IMPα is deemed to be “autoinhibited” with only low affinity for NLSs due to binding of its IBB in the NLS-binding pocket of IMPα [15]; this is essential to facilitate cargo release in the nucleus [11,16] after IMPβ1 is dissociated from the transport complex upon binding of the monomeric guanine nucleotide-binding protein Ran in its activated GTP-bound form [10,11,13]. Dysregulation of nucleocytoplasmic transport can impact a range of cellular processes [9,15,16], with the inhibition of nuclear transport holding great potential for therapeutic intervention [17,18,19,20].

IMPs have been used in high-throughput screens (HTS) to identify small molecules that target IMPα/β-dependent nuclear import of viral proteins central to infection for Human Immunodeficiency Virus, dengue, and Venezuelan Equine Encephalitis Virus [19,20,21,22,23,24]. Importantly, a number of these, including ivermectin, which is of current interest with respect to SARS-CoV-2, have been shown to limit viral infection by preventing the host IMPα recognition/nuclear localization of viral proteins in infected cells [19,20,22,23,24,25,26,27,28,29,30]. Clearly, inhibitors of nuclear transport machinery have considerable therapeutic potential [17,18,19,20,26,31].

The fact that *P. falciparum* and *T. gondii* divide rapidly within the human host implies a need for efficient nuclear transport systems [32], raising the possibility that the nuclear trafficking pathways of apicomplexans could serve as targets for therapeutics to limit infection. Importantly, in this context, both *P. falciparum* and *T. gondii* have a single genomic copy of IMPα [9] that is essential [33,34]. *P. falciparum* IMPα (PfIMPα) is known to bind to a “classical NLS” identified in *P. falciparum* trimethyl guanosine synthase 1 (TGS1) that methylates the terminal phosphate groups of spliceosomal RNAs [35,36], yet appears to show a unique lack of autoinhibition [37], which as indicated above, is central to the function of mammalian IMPαs [15,16]. Apicomplexan IMPα would appear to be an intriguing prospect as a target for inhibitors to limit diseases caused by *P. falciparum* and *T. gondii*.

Here we describe, for the first time, a high-throughput screen (HTS) to identify small molecule inhibitors of PfIMPα: TGS1-NLS interaction using AlphaScreen technology [21,22,23,38]. In total, 13 small molecules of interest were identified as hits and screened for their ability to inhibit interaction between mammalian IMPα and *T. gondii* IMPα (TgIMPα) and the well-characterized Simian Virus 40 T-antigen (SV40 T-ag) NLS, with the compound Bay 11-7085 showing high selectivity for PfIMPα. Thermostability assays were used to confirm the direct binding of Bay 11-7085 and other compounds to PfIMPα, and their ability to inhibit growth by both *P. falciparum* and *T. gondii* was also confirmed. The results establish the principle that apicomplexan IMPα is a viable target for drug discovery to combat malaria as well as toxoplasmosis.

## 2. Materials and Methods

### 2.1. Plasmid Construction

All restriction enzymes were obtained from Thermo Fisher Scientific (Waltham, MA, USA), and gene amplification was performed using the KAPA HiFiTM PCR kit (Kapa Biosystems, Wilmington, MA, USA) unless otherwise indicated. Primers were procured from Integrated DNA Technologies (IDT, Coralville, IA, USA). The integrity of all plasmid constructs was confirmed by DNA sequencing.

To generate a TgIMPα C-terminal His-tagged bacterial expression construct, the TgIMPα coding sequence was PCR-amplified from cDNA and inserted between the NcoI and HindIII restriction sites of plasmid vector pET28a. Primers used to amplify the TgIMPα gene were (Forward: 5′ CATGCCATGGAGCGCAAGTTGGCCGATC 3′, Reverse: 5′ TCCCAAGCTTCTGGCCGAAGTTGAAGCCTC 3′; restriction sites underlined).

To generate a C-terminal glutathione S-transferase (GST) fusion protein expression construct in plasmid vector pET28a in place of the C-terminal hexa-Histidine tag, the GST coding sequence was PCR amplified from plasmid pGEX-6p1 and inserted into the NheI and HindIII restriction sites of pET28a. The primers used to amplify the GST gene sequence were (Forward: 5′ CTAGCTAGCTCCCCTATACTAGGTTATTGG 3′ and Reverse: 5′ CCCAAGCTTTCAGTCACGATGCG 3′; restriction sites underlined). *P. falciparum* (PlasmoDB ID: PF3D7_0812400) and *T. gondii* IMPα (ToxoDB ID: TGGT1_252290) with C-terminal hexa-Histidine tags were subcloned as PCR products into the NcoI and Nhe I sites of the pET28a-C GST vector. The primers used for PCR amplification were as follows: (PfIMPα: Forward: 5′ CATGCCATGGATAGGAGAATAGAAGCTAG 3′; Reverse: 5′ CTAGCTAGCGTCAAATGTAAAAT-CCTTATTTAAAAC 3′; TgIMPα: Forward: 5′ CATGCCATGGAGCGCAAGTTGGCCG 3′; Reverse: 5′ CTAGCTAGCCTGGCCGAAGTTGAAGCC 3′; restriction sites underlined), with PfIMPα amplified from the previously described hexa-Histidine-tagged expression construction [37], and TgIMPα amplified from a construct generated in identical fashion.

### 2.2. Protein Expression, Purification, and Use in AlphaScreen Assay

PfIMPα, TgIMPα, *Mus musculus* IMPα (MmIMPα), ΔIBBMmIMPα, and β1 (MmIMPβ) GST fusion proteins were expressed and purified essentially as previously [21,22,23]. His-tagged TGS1-NLS-GFP, PfIMPα and TgIMPα proteins were purified using Ni^2+^-affinity chromatography as described [37]. His-tagged SV40 T-ag-NLS-GFP was purified as previously [38]. Biotinylation of GST-tagged proteins was carried out using the Sulfo-NHS-Biotin reagent (Pierce, Rockford, IL, USA) as described previously [21]. AlphaScreen binding assay was performed as described previously [21,22,23,24,27,28,29,30,38].

### 2.3. HTS

HTS in the AlphaScreen system was performed robotically as previously [21,22,23] to identify inhibitors of PfIMPα: TGS1 NLS-GFP interaction from the MMV Pathogen Box Chemical Library (400 compounds; Medicines for Malaria Venture, Geneva, Switzerland) and Library of Pharmacologically Active Compounds (LOPAC1280; Sigma-Aldrich, St Louis, MO, USA; 1280 compounds). Then, 10 nM biotinylated PfIMPα and 60 nM TGS1-NLS-GFP were used with library compounds, screened at 10 µM final concentration.

Briefly, 5 μL of each compound in DMSO was added to quadruplicate wells of a 384-well plate along with appropriate controls including DMSO alone and ivermectin, as previously [23], using a JANUS Modular Dispense Technology (MDT) robotic system (PerkinElmer, Waltham, MA, USA). This was followed by successive 7 and 5 μL additions, respectively, of PfIMPα (10 nM) and TGS1-NLS-GFP (60 nM) or PBS (“no bait” control), respectively, and 30 min incubation at room temperature. AlphaScreen acceptor beads (1/40 dilution of Ni-NTA beads and 6.25 % BSA in PBS) were then added to each well using a Multidrop liquid dispenser (Thermo Fisher Scientific, Waltham, MA, USA) and the plates incubated for 90 min at room temperature in the dark. Then, 4 µL of the AlphaScreen donor beads (1/40 dilution of GSH. acceptor beads in PBS) was finally added to each well using a Multidrop, followed by a further incubation of 2 h at room temperature in the dark.

Plates were read for an Alphascreen signal on an Envision plate reader (PerkinElmer, Waltham, MA, USA), with signals from the negative control wells (no bait control) on each plate subtracted. Compounds showing more than 70% inhibition (DMSO negative control = 100%) were tested to exclude false-positives by counter-screening in the system using 5 nM hexa-His-Biotin in place of hexa-His-tagged and biotinylated proteins [21,22,23].

### 2.4. Thermostability Assay (TSA)

The effect of inhibitory compounds on IMPα thermostability was tested, as previously, using the fluorescent dye SYPRO Orange (Thermo Fisher Scientific, Waltham, MA, USA) and the Qiagen Rotor-Gene Q6 plex instrument programmed in the melt curve mode [28,30]. Increasing concentrations of compound in 0.5 µL DMSO were added to 24.5 µL recombinant protein (2 µM) in phosphate-buffered saline (PBS). Next, 1 µL of SYPRO Orange dye was added, the reaction mixture heated at 0.5 °C/min from 27 °C to 90 °C, and the fluorescence intensity caused by the SYPRO Orange binding to the proteins monitored (excitation/emission: 530/555 nm). T_m_ is the temperature at which 50% of the protein is unfolded.

### 2.5. P. falciparum Culture and Growth Inhibition Assay

Red blood cells (RBCs) were derived from blood from volunteers (approval from the Institute Ethics Committee, IIT Bombay, Mumbai, India) by density gradient centrifugation. Aliquots of cryopreserved *P. falciparum* 3D7 strain in human red blood cells (RBCs—3% hematocrit, <5% parasitemia) were thawed and cultured using RPMI (Roswell Park Memorial Institute) 1640 medium supplemented with 0.5% Albumax (Gibco^TM^, Waltham, MA, USA), 50 mg/L hypoxanthine (Sigma-Aldrich, St Louis, MO, USA), 2 g/L D-glucose (Sigma-Aldrich, St Louis, MO, USA), 2 g/L sodium bicarbonate (Sigma-Aldrich, St Louis, MO, USA), and 56 mg/L of gentamicin (Abbott, Chicago, IL, USA) at 37 °C in 5% CO_2_ in a humidified incubator according to the standard procedures [39]; suspension cultures were maintained at 3% hematocrit and <5% parasitemia, by adding a fresh medium daily and a regular addition of fresh human RBCs.

Before growth inhibition assays, cultures were synchronized using 5% D-sorbitol (Sigma-Aldrich, St Louis, MO, USA) predominantly to obtain the ring stage of the *P. falciparum* life cycle. The test compounds auranofin, Bay 11-7085, CAPE (all from Sigma-Aldrich, St Louis, MO, USA) and Zoxazolamine-MMV003270 (MMV, Geneva, Switzerland) were made up at a stock concentration of 10 mM in DMSO and diluted in RPMI as required for growth inhibition studies or IC50 analysis, with dihydroartemisinin (gift from IPCA Laboratories, Mumbai, India) used as a control.

Growth assays in the absence and presence of inhibitors were performed using the Histidine–Rich Protein 2 (HRP2) sandwich horseradish peroxidase-linked immunosorbent assay to measure HRP2 protein levels as an indicator of growth [40,41]. Briefly, 25 µL of compound was added to 96-well microculture plates (Eppendorf, Hamburg, Germany) of 0.25% (with fresh RBCs), and added at a hematocrit of 3%. The plates were then incubated for 72 h at 37 °C in a humidified incubator, sparged with 5% CO_2_. 72 h later, the plates were subjected to freeze–thaw to achieve hemolysis, and samples diluted 200-fold in H_2_O in transferring to another plate. A day prior to analysis, 96-well ELISA plates (Sigma-Aldrich, St Louis, MO, USA) were coated overnight at 4 °C with 100 µL of 1 μg/mL IgM capture antibody MPFM-55A (Immunology Consultants Laboratory, Portland, OR, USA) specific for *P. falciparum* HRP2. After blocking for 2 h at room temperature with 2% BSA in PBS, diluted samples were added to the wells, followed by incubation for 2 h, then the plates were washed three times with 0.05% Tween 20 in PBS. Next, 100 µL of the secondary antibody MPFG-55P (Immunology Consultants Laboratory, Portland, OR, USA) conjugated with horseradish peroxidase (0.25 μg/mL in PBS with 2% BSA) was then added to each well, and the plates incubated for 1 h at room temperature, prior to being washed three times, and 100 µL 3,3′, 5,5′ tetramethylbenzidine substrate (BD Biosciences, San Jose, CA, USA) being added. After 10 min incubation in the dark, 50 µL of 1 M H_2_SO_4_ was added and the absorbance was read at 450 nm on a Multiscan™ FC Microplate Spectrophotometer (Thermo Fisher Scientific, Waltham, MA, USA).

### 2.6. T. gondii Tachyzoite Culture and Growth Inhibition Assay

*T. gondii* RH strain expressing the luciferase reporter (RH-Fluc) was derived to assess the effect of the inhibitors on growth. Briefly, plasmid pCTG-EGFP [42] was modified by replacing the coding sequence of EGFP with that of the firefly luciferase reporter, using restriction enzymes BglII and PstI (Thermo Fisher Scientific, Waltham, MA, USA). The resultant plasmid, pCTG-Fluc, expressing firefly luciferase constitutively under the control of the *T. gondii* TubulinA promoter, was electroporated into the *T. gondii* RH strain [43,44] using a Bio-Rad GenePulser Xcell system (1500 V, 50 Ω and 25 µF) along with restriction enzyme NotI to initiate RE-Mediated Integration (REMI) of the linearized plasmid into the genome [45,46]. The resultant stable *T. gondii* line expressing the luciferase reporter was selected for resistance to 20 µM chloramphenicol (encoded by the plasmid pCTG), and dilution cloning [47] was performed to ultimately identify the clone (RH-Fluc) with the highest luciferase activity (c. 4 × 10^7^ RLU from 10^7^ parasites) used for growth assays. *T. gondii* tachyzoites were maintained and cultured at 37 °C in 5% CO_2_ in a humidified incubator in primary human foreskin fibroblasts (HFF, ATCC) [47,48]; HFF cells were maintained in Dulbecco’s modified Eagle medium (DMEM) (Gibco^TM^, Waltham, MA, USA) supplemented with 3.7 g/L sodium bicarbonate and 2.38 g/L HEPES, 10% Cosmic Calf serum (Hyclone^TM^, Logan, UT, USA) and 20 mg/L gentamicin [47,48].

Growth inhibition was assessed by luminescence measurements as described [49,50]. Briefly, 100 μL of culture medium with or without inhibitors was added to confluent monolayers of HFF cells grown in 96-well treated culture plates (Eppendorf, Hamburg, Germany), followed by 100 µL of DMEM containing 5000 parasites. After 48 h, 150 µL of the culture media was discarded from each well without aspirating any parasites, and 10 µL lysis buffer was added to lyse the parasites, followed by 50 μL of 2× luciferase assay reagent (Promega, Madison, WI, USA). Luminescence was measured directly for 10 s using a Varioskan™ LUX multimode microplate reader (Thermo Fisher Scientific, Waltham, MA, USA).

### 2.7. MTT Assay for Host Cell Cytotoxicity

The MTT assay was used to measure the cytotoxicity of the small molecules against the HFF cells as described [51]. Briefly, freshly confluent HFF cells in a 96-well culture plate were treated with increasing concentrations of compounds for 48 h, prior to the MTT assay.

### 2.8. Statistical Analysis

Four parameter dose response curves were fitted using non-linear regression analysis in GraphPad Prism 9.2.0 (San Diego, CA, USA) using the formula: y = a + ((b − a)/(1 + 10^(Log(c) − x) × d)), where a is the minimum asymptote, b is the maximum asymptote, c is the half-maximal inhibitory concentration value (IC_50_) and d is the slope at the steepest part of the curve (the Hill slope).

## 3. Results

### 3.1. Optimisation of AlphaScreen Binding Assay for HTS

**Figure 1:**
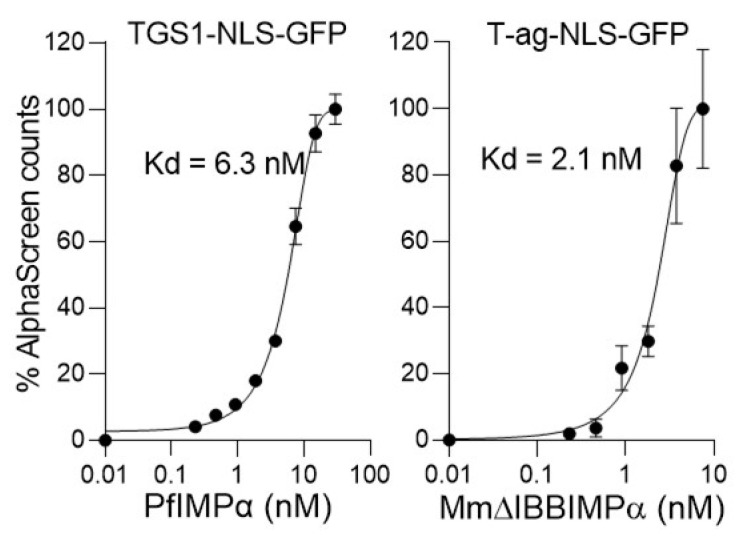
PfIMPα shows high affinity binding to the TGS1 NLS, comparable to that for MmΔIBBIMPα. AlphaScreen technology was used to determine the K_d_ value of NLS-GFP (30 nM) binding to biotinylated-GST-IMPαs (5 nM). Data points in the figures represent the AlphaScreen signal for NLS-GFP and IMPα interaction from a single typical experiment from a series of three independent experiments. Pooled data showed K_d_ values of 6.5 ± 0.2 and 2.6 ± 0.5 nM (mean ± SEM, *n* = 3) for PfIMPα:TGS1-NLS and MmΔIBBIMPα:T-ag-NLS, respectively.

HTS was performed using a roboticised AlphaScreen system, as previously [21,22,23], to identify small molecules inhibiting the interaction of PfIMPα and the *P. falciparum* TGS1 NLS [37]. Unlike mammalian IMPα, full-length PfIMPα lacks autoinhibition, not requiring IMPβ1 for high affinity binding to NLSs [37]. This is illustrated in Figure 1, where NLS-binding by full-length PfIMPα is compared to that for MmΔIBBIMPα, a form of *M. musculus* IMPα deleted for the autoinhibitory IBB domain. PfIMPα shows high-affinity binding (dissociation constant, K_d_, of 6.5 nM) to the TGS1 NLS, comparable to that for MmΔIBBIMPα binding to the well-characterized simian virus SV40 large tumor antigen (T-ag) (Figure 1; see legend). Based on these results and further optimization in the robotic system (not shown), the concentrations of PfIMPα and TGS1-NLS-GFP selected for use in HTS were fixed to 10 and 60 nM, respectively, giving c. 70% maximal AlphaScreen signal, with the possibility to identify agents either inhibiting or enhancing the signal [21,22,23].

### 3.2. Library Screening for Inhibitors of the Interaction between PfIMPα and TGS1-NLS

**Figure 2:**
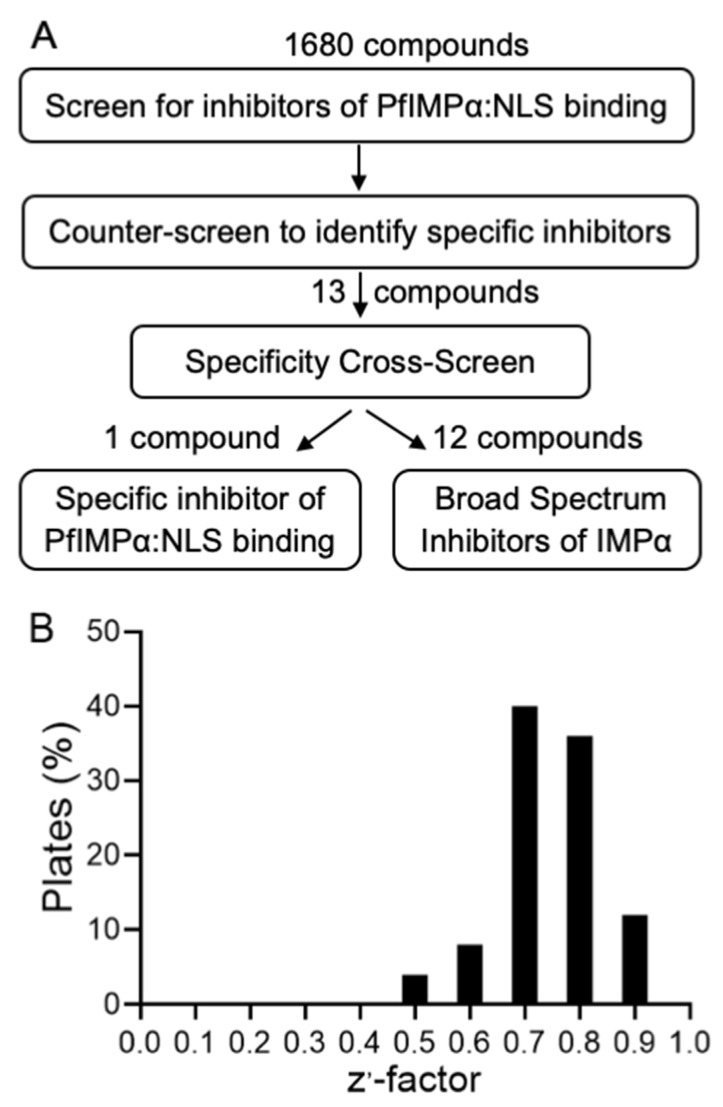
HTS to identify inhibitors of PfIMPα:NLS binding. (**A**) Schematic showing the strategy used to identify inhibitors of PfIMPα:NLS binding in the AlphaScreen system from the LOPAC and Pathogen Box libraries; the numbers of compounds at different stages of the screening/counter-screening process are indicated. (**B**) Plot of distribution of Z′ factors for the HTS, calculated as previously [21,22,23,52], as a % of the number of plates.

Using the assay optimized in Section 3.1, HTS was performed essentially, as previously [23], to identify inhibitors of the interaction between PfIMPα and the TGS1-NLS (see Figure 2A), using the LOPAC and Pathogen Box chemical libraries; compounds were screened in quadruplicate, together with appropriate controls as previously [23]. The assay’s robustness was confirmed by the Z’ factor (values > 0.5 across all plates, with a median of 0.75—see Figure 2B) [52]. Compounds inhibiting the maximum signal compared to the DMSO control by >70% were counter-screened as previously for interference with the AlphaScreen assay, where biotinylated and His-tagged proteins were replaced with biotinylated-His, which generated a strong AlphaScreen signal [21,22,23]. Ultimately, 13 compounds were selected for further analysis, as outlined in Figure 2A (see below). These included auranofin/MMV688978, which was identified as a strong hit from both the LOPAC and Pathogen Box libraries.

### 3.3. Cross-Screening to Identify Selective Inhibitors of PfIMPα:TGS1–NLS Binding

To determine the extent to which the hit compounds may be selective for PfIMPα, we compared their ability to inhibit NLS binding by PfIMPα, mammalian non-autoinhibited MmIMPα (MmΔIBBIMPα) as well as the MmIMPα/β heterodimer, and the apicomplexan *T. gondii* IMPα (TgIMPα). The results are summarized in Table 1; all compounds inhibit PfIMPα by 70% or more, with a cut-off of 50% inhibition or higher used to assess selectivity. “General inhibitors” thus block interactions between all of the four IMP-NLS interactions by at least 50%, whereas “selective inhibitors” inhibit a subset of the IMP-NLS interactions by 50% or more.

At 10 μM, 11 of the compounds showed stronger inhibition of the PfIMPα:NLS interaction than of the MmΔIBBIMPα:NLS or TgIMPα:NLS interactions (Table 1), indicating overall selectivity towards PfIMPα. Daphnetin was an exception in that it inhibited PfIMPα and MmΔIBBIMPα to the same extent, whilst caffeic acid phenethyl ester (CAPE) showed higher inhibition of MmΔIBBIMPα than PfIMPα (Table 1). A number of compounds (Table 1A) showed >50% inhibition of all four IMP-NLS binding interactions, with the implication that they are “general” inhibitors of IMPα, even in the context of the IMPα/β heterodimer. The other inhibitors showed various degrees of selectivity, inhibiting one or a subset of the IMP-NLS interactions, although not all.

Bay 11-7085 appeared to be highly selective for PfIMPα (75% inhibition) with 0–14% inhibition of the other IMPαs and the MmIMPα/β heterodimer (Table 1). MMV030734 showed a similar trend, however showed 68% inhibition of MmIMPα/β; interestingly, MMV030734, MMV676512 and (±)-epinephrine HCl were the most potent inhibitors of PfIMPα (≥98% inhibition), all inhibited TgIMPα and MmΔIBBIMPα to <55%, and all inhibited MmIMPα/β (in the case of MMV676512 and (±)-epinephrine HCl to ≥95%). Finally, based on the 50% inhibition criterion, CAPE was the only inhibitor that inhibited all three IMPαs, yet did not inhibit MmIMPα/β (Table 1).

**Figure 3:**
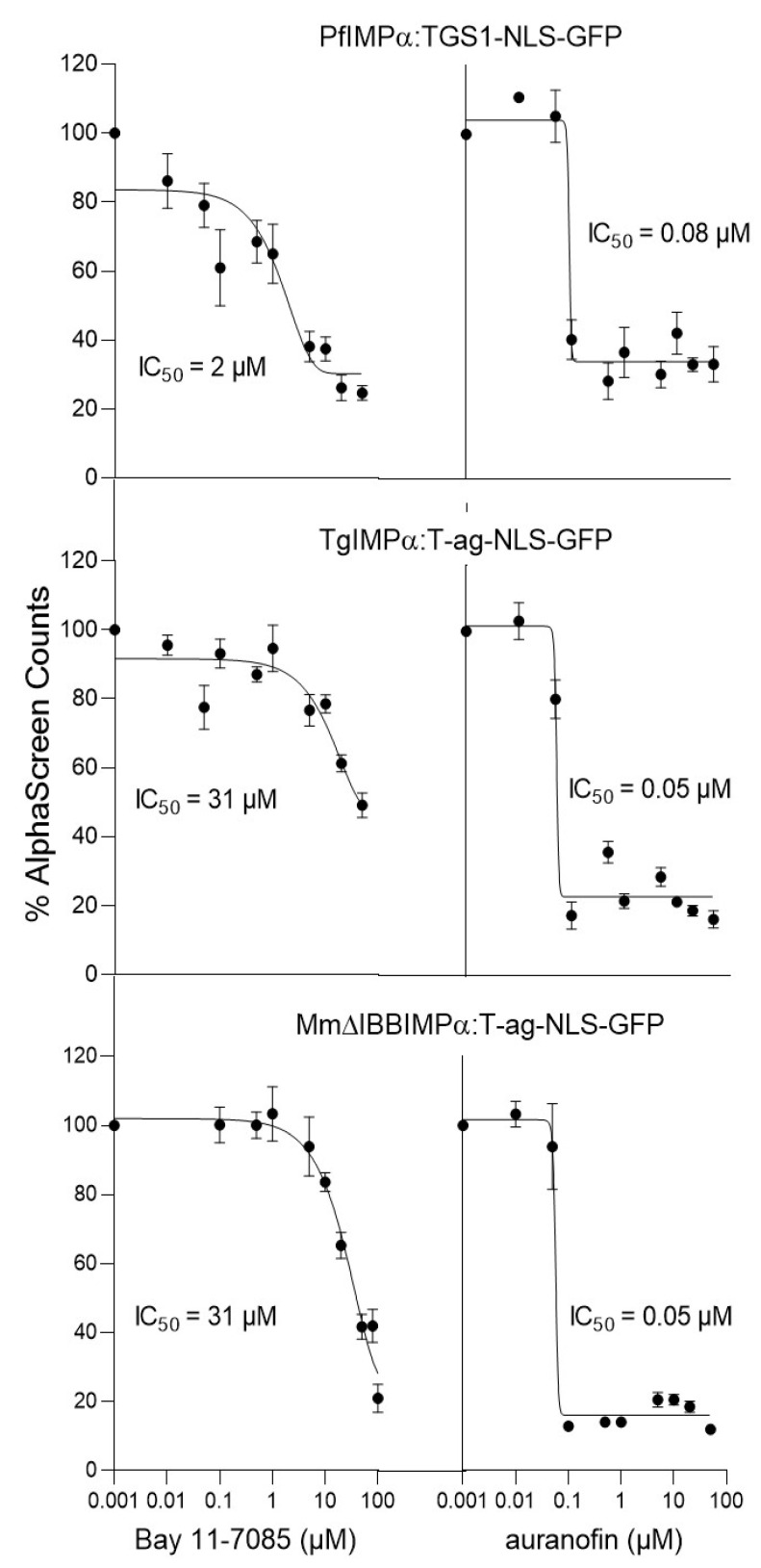
Bay 11-7085 specifically inhibits PfIMPα-TGS1-NLS interaction at low micromolar concentration, whereas auranofin is a general inhibitor of IMPα:NLS interactions. AlphaScreen technology was used to determine the IC_50_ for inhibition by Bay 11-7085 and auranofin binding of IMPαs (5 nM) to NLS (30 nM). Data represent the mean ± SEM (*n* = 4) from a single experiment, from a series of three independent experiments (see Table 3 for pooled data).

Based on the cross-screen analysis, auranofin, Bay 11-7085, CAPE, MMV003270 were subjected to IC_50_ analysis for the three IMPα:NLS interactions (see Table 2), and the results generally indicated low μM values and support the conclusions from Table 1 with respect to selectivity. The results for Bay 11-7085, for example, indicate c. 16 and 12-fold higher IC_50_ values for inhibition of the TgIMPα-NLS and ΔIBBMmIMPα-NLS interactions, respectively, (31 and 23 μM values) compared to that for the PfIMPα-TGS-NLS interaction (2 µM) (Figure 3,Table 3), supporting the idea that Bay 11-7085 is highly selective for PfIMPα. Auranofin, in contrast, showed robust inhibition (IC_50_ values of 60–80 nM) of all three IMPα:NLS interactions (Figure 3), consistent with it being a strong general inhibitor; CAPE (IC_50_ values of 0.7–2.9 μM) and MMV0033270 (IC_50_ values of 200–400 nM) showed a similar inhibitory profile, although they were not as potent (see Table 3).

### 3.4. Bay 11-7085 and Auranofin Appear to Bind Directly to IMPα

**Figure 4:**
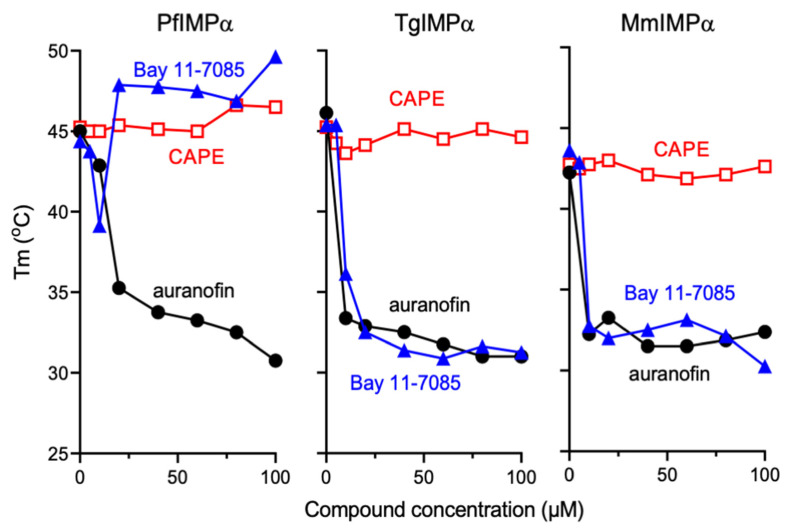
Bay 11-7085 and auranofin appear to bind directly to IMPαs as indicated by thermostability analysis. The indicated His6-tagged IMPα proteins were subjected to thermostability analysis in the presence of increasing concentrations in the indicated compounds. Results are from a single experiment, representative of three independent experiments.

A thermostability assay (TSA) was used as previously [28,30] to examine direct binding of the compounds to the different IMPαs. PfIMPα, TgIMPα and MmIMPα were all similar in showing stability up to c. 43–45 °C in the absence of the inhibitor compounds (see also [28,30]). Although MMV003270 could not be sourced in sufficient amounts for the assay, we tested the PfIMPα selective inhibitor Bay 11-7085, the general inhibitor auranofin, and the IMPα selective inhibitor CAPE. The latter did not elicit any marked change in stability for any of the IMPαs up to 100 µM, with the possible exception being for PfIMPα, where concentrations of 75 µM or higher elicited a slight increase of 2 °C in thermostability (to 47 °C—see Figure 4 left panel); both Bay 11-7085 and auranofin, in contrast, had marked effects on thermostablity of all three IMPαs, consistent with the idea that they bind directly to the IMPαs, thereby altering IMPα structure and thereby thermostability. Auranofin at concentrations as low as 5 μM appeared to reduce thermostabililty in the case of all three IMPαs (stable only up to 32–35 °C) by 12–15 °C (Figure 4). Bay 11-7085 at 10–20 µM similarly reduced the thermostability (9–15 °C) of both TgIMPα and MmIMPα (stable only up to 30–36 °C—Figure 4 middle and right panels). Strikingly, however, it appeared to have a biphasic effect on PfIMPα, concentrations up to 10 µM reducing stability by 6 °C (to 39 °C), and concentrations higher than 20 µM increasing stability by 9–11 °C (to 48–50 °C—Figure 4 left panel). Whether these effects could indicate the more than one binding mode/site for Bay 11-7085 on PfIMPα will require detailed experimental investigation in the future, however, the clear implication is that Bay 11-7085 binds to PfIMPα in a mode quite distinct to that of auranofin, and quite distinct to the effect of its binding on the other IMPαs; this may well be the basis of the observations from the binding assays (Figure 3, Table 2) that show that although they are able to inhibit NLS binding by both MmIMPα and TgIMPα, they do so only at concentrations 12-16 times higher than those required to inhibit NLS binding by PfIMPα.

### 3.5. PfIMPα Inhibitors Limit Proliferation of P. falciparum and T. gondii In Vitro

**Figure 5:**
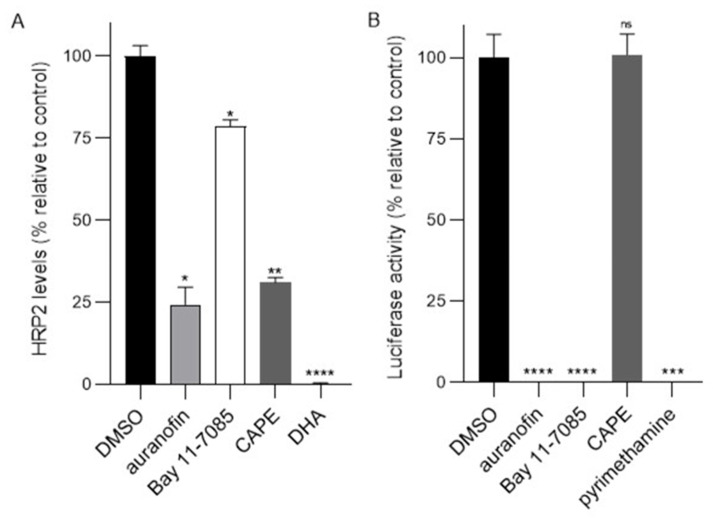
Results for the initial screening of selected hit compounds for antimalarial activity towards *P. falciparum* (**A**) and *T. gondii* (**B**). (**A**) *P. falciparum* cultures (0.25% parasitemia) were treated with 10 µM concentration of the indicated compounds for 72 h, after which the HRP2-based sandwich ELISA was used to measure the HRP2 levels determined by optical density. Results are from a single typical experiment performed in duplicate (SD shown), representative of a series of three independent experiments. *, *p* < 0.05; **, *p* < 0.01; ****, *p* < 0.0001. (**B**) HFF cells infected with RH-Fluc *T. gondii* parasites were incubated with 10 µM c of the indicated compounds for 48 h, after which parasite growth was measured using a luciferase assay. Results are from a single typical experiment performed in duplicate (SD shown), representative of a series of three independent experiments. ***, *p* < 0.001; ****, *p* < 0.0001; ns, not significant.

To begin to assess formally the importance of NLS recognition by *P. falciparum* and *T. gondii* IMPα in a physiological context, we screened the compounds against both parasites at a single concentration (10 µM). For *P. falciparum,* we employed the standard HRP2-based (histidine-rich protein 2) ELISA, with the clinically prescribed drug dihydroartemisinin (DHA) as a positive control against *P. falciparum* at 10 µM [40,41,53]. Initial analysis showed that auranofin and CAPE showed >50% inhibition at 10 µM (Figure 5A); Bay 11-7085 showed c. 20% inhibition. The basis for Bay 11-7085′s relatively low activity in this assay is unclear, however, it may relate to the limited ability of the compound to be taken up by the malarial parasite, or low stability (see Section 4).

For *T. gondii*, we used tachyzoites constitutively expressing luciferase (see Section 2.6), with the clinically prescribed toxoplasmosis drug pyrimethamine used as a positive control [54]. A > 50% reduction in luciferase activity, indicative of growth inhibition, was effected at 10 μM by auranofin and Bay 11-7085, however, CAPE showed only 5% inhibition (Figure 5B). As for Bay 11-7085 above, the basis for the low activity in this assay is unclear although it may relate to the poor uptake of the compound by the tachyzoites, or reduced stability (see Section 4); interference with the luciferase chemistry seems unlikely (e.g., see [55]).

**Figure 6:**
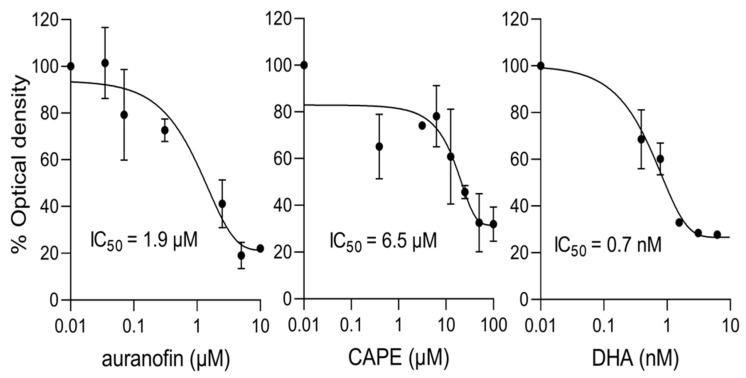
Auranofin and CAPE inhibit *P. falciparum* parasites grown in culture at low μM concentrations. *P. falciparum* cultures (0.25% parasitemia) were treated with increasing concentrations of the indicated compounds for 72 h, after which the HRP2-based sandwich ELISA was used to measure the HRP2 levels, determined by optical density. Results shown are from a single typical experiment performed in duplicate (SD shown), representative of a series of three independent experiments (see Table 3 for pooled data).

**Figure 7:**
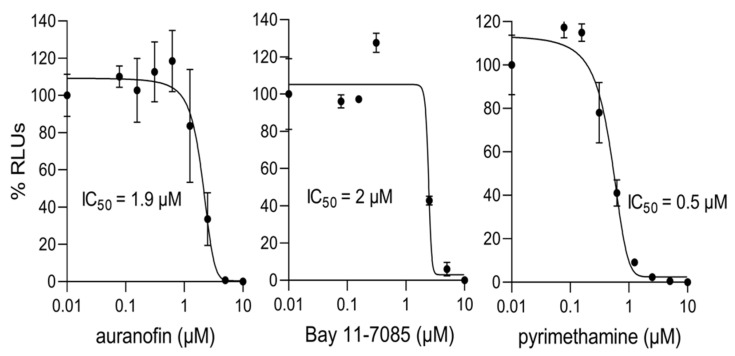
Auranofin and Bay 11-7085 inhibit *T. gondii* in vitro at low μM concentrations. HFF cells infected with RH-Fluc parasites were incubated with increasing concentrations of the indicated compounds for 48 h. Parasite growth was measured using a luciferase assay. Results shown above are from a single typical experiment performed in duplicate (SD indicated), from a series of three independent experiments (see Table 3, right column, for pooled data).

Detailed IC_50_ analysis for activity against *P. falciparum* was performed for auranofin and CAPE, with results indicating values in the low μM range (Figure 6, Table 3 central column); clearly, both have robust antimalarial activity. In parallel, detailed IC_50_ analysis were similarly carried out for Bay 11-7085 and auranofin with respect to *T. gondii*; results indicated robust antiparasitic activity, with IC_50_ values in the low µM concentration range (Figure 7; Table 3 right column).

To confirm that the above effects were not attributable to cytotoxicity effected by the compounds on the HFF cells used in the *T. gondii* infectious system, HFF cells were treated with Bay 11-7085 and auranofin and viability monitored using the MTT assay, as previously. Auranofin and Bay 11-7085 showed CC_50_ values for HFF cells of five and eight μM, respectively, (Figure S1, Table S1), corresponding to selectivity index values of two (auranofin) and three (Bay 11-7085) (Table S1). What is clear is that at concentrations < c. 5 µM, cytotoxicity is essentially absent; since parasite killing at 2 µM or less is substantial (≥60%—see Figure 7 left panels) in the case of both, auranofin (malaria and toxoplasmosis) and Bay 11-7085 (toxoplasmosis) represent interesting prospects for future development as antiparasitic agents.

## 4. Discussion

Emerging drug resistance on the part of *P. falciparum* and *T. gondii* reinforces the urgent need to find new drugs to combat malaria and toxoplasmosis. Importantly in this context, this is the first study to use HTS to identify small molecules that target PfIMPα. In particular, we screened for compounds able to inhibit PfIMPα recognition of a *P. falciparum* NLS (from TGS1, involved in methylation of *P. falciparum* splicesome RNAs). Through cycles of counter screening, selectivity testing, IC_50_ analysis, and antiparasitic testing, it proved possible to identify several small molecules that have the ability to both inhibit PfIMPα:NLS binding, and limit the growth of *P. falciparum* and/or *T. gondii*, as we show here for the first time. Importantly, this study validates IMPα-dependent nuclear protein import as a target for therapeutic intervention in the case of apicomplexan parasites, consistent with the fact that both parasitic organisms have a single, essential IMPα gene [5,33,34].

Of the various hit compounds analyzed, Bay 11-7085 was unique in being a selective inhibitor of PfIMPα (12-16-fold lower IC_50_ value), compared to both TgIMPα and MmIMPα in IMPα:NLS binding assays. Further, TSA analysis showed that binding of this compound has differential effects on PfIMPα compared to the other IMPαs; firstly, binding of Bay 11-7085 to PfIMPα increased its thermostability, in contrast to both TgIMPα and MmIMPα where binding decreases thermostability (Figure 4). Further, TSA analysis for Bay 11-7085 indicated a biphasic effect for PfIMPα (Figure 4 left), where lower concentrations appeared to destabilize PfIMPα (in similar fashion to Bay 11-7085′s effects throughout on TgIMPα and MmIMPα; see Figure 4 middle and right), although higher concentrations (>20 μM) led to a marked stabilization of the protein. The results imply that Bay 11-7085 likely binds a site(s) onto the PfIMPα that is distinct to the site(s) it binds onto the TgIMPα and MmIMPα IMPα, consistent with the fact that the amino acid similarity between PfIMPα and TgIMPα and PfIMPα and MmIMPα, respectively, is 54 and 41% (with homology between TgIMPα and MmIMPα 43%). The distinct effects of the binding of Bay 11-7085 to PfIMPα are thus likely due to key sequence differences between the various IMPαs. Molecular docking and crystallographic studies of PfIMPα in the absence or presence of Bay 11-7085 should help confirm this, as well as provide more detailed information about the mechanisms of the binding of these small molecules, and help guide future efforts to develop small molecules specifically targeting “hot spot residues” of PfIMPα.

Surprisingly, although Bay 11-7085 appeared to be a selective inhibitor of PfIMPα binding to *P. falciparum* TGS1- NLS in vitro, it was not a potent inhibitor of *P. falciparum* parasites in culture (Figure 5A); in contrast, it exhibited strong inhibition of *T. gondii* tachzoites (Figure 5B; Figure 7 middle panel). This suggests that there may be differences in the uptake and/or catabolism of the compound in the case of *P. falciparum* parasites and *T. gondii* tachzoites, which is a limitation in terms of considering Bay 11-7085 as a potential anti-malarial agent in the future. Bay 11-7085 is a known irreversible inhibitor of Nuclear Factor-κB (NF-κB) [56]; although antiparasitic activity has not previously been reported for Bay 11-7085, a closely related structural analogue Bay 11-7082 has been shown to inhibit the growth of *T. gondii* tachyzoites [49]. This is consistent with the Bay 11-7085/7082 scaffold having inhibitory activity towards *T. gondii*. In fact, as shown here for the first time, Bay 11-7085 can kill *T. gondii* tachyzoites at low μM concentration, with a selectivity index of three; Bay 11-7085 and its structural analogues thus represent a viable starting point for drug development to combat *T. gondii*, and potentially also *P. falciparum*.

In terms of the less selective hit compounds emerging from our screen, auranofin (MMV688978) was the most potent, showing IC_50_ values in the nM range for PfIMPα:NLS binding. Auranofin exhibits reactivity for the thiol group of proteins [57] and is a potent inhibitor of thioredoxin reductase enzymes, which may be the basis for its apparent action on IMPα from PfIMPα. TSA analysis implies that auranofin can destabilize (reduce the thermostability of) all three IMPα proteins examined here. Auranofin has previously been shown to possess activity against *P. falciparum* [58], as well as cultured *T. gondii* [59], and we confirm this here for both parasites. Since auranofin is FDA-approved for human use in gold-conjugated form to treat rheumatoid arthritis [57], its apparent effect on apicomplexan IMPα documented here, together with its robust antiparasitic effects against *P. falciparum* and *T. gondii*, indicate its potential for future investigation as an antiparasitic for malaria and toxoplasmosis.

In summary, this is the first HTS to target PfIMPα binding to NLS-containing proteins. The fact that several hit compounds possess antiparasitic activity with low host cell cytotoxicity, validates apicomplexan IMPα as a therapeutic target, and opens the way both for larger screens using strategies similar to those described here, and for future investigation into the candidate hit molecules identified here in terms of the molecular details of the host-pathogen interaction. The latter is a priority for future work in this laboratory.

## Figures and Tables

**Table 1:** Selectivity of PfIMPα inhibitors.

Hit Compound ^x^	% Inhibition of IMPα-NLS Interaction *			
	PfIMPα	TgIMPα ^#^	MmΔIBBIMPα	MmIMPα/β
**(A) General Inhibitors**				
MMV688978 (auranofin)	94	90	91	100
Daphnetin	81	70	81	83
Chelerythrine Cl	81	67	88	94
AC-93253 iodide	82	72	67	62
MMV003270	92	83	82	NT ^$^
6-Fluoronorepinephrine HCl	77	67	60	51
**(B) Inhibitors Showing Selectivity**				
**(B1) Selective for PfIMP** **α**				
Bay 11-7085	75	0	9	14
**(B2) Selective for IMP** **αs**				
Caffeic acid phenethyl ester	84	62	92	14
**(** **B3) Others**				
MMV030734	99	44	43	68
MMV024937	73	26	70	24
(−)-Epinephrine bitartrate	85	23	34	94
(±)-Epinephrine HCl	98	24	35	95
MMV676512	100	48	54	100

* Results represent the average percentage (%) inhibition of the AlphaScreen signal relative to the 1% DMSO control tested in quadruplicate, with compounds at 10 μM and protein concentrations as follows: PfIMPα (10 nM):TGS1-NLS-GFP (60 nM), ΔIBBMmIMPα (5 nM):T-ag-NLS-GFP (30 nM), MmIMPα/β (5 nM):T-ag-NLS-GFP (30 nM) and TgIMPα (5 nM):T-ag-NLS-GFP (30 nM). ^x^ All compounds inhibit PfIMPα:NLS > 70%; a cut-off of 50% inhibition is arbitrarily used for the working categories in A and B (highlighted by blocking). ^#^ K_d_ for TgIMPα (5 nM):T-ag-NLS-GFP (30 nM) binding without inhibitors is 3.5 ± 1 nM (*n* = 3). ^$^ NT, not tested.

**Table 2:** Summary of the IC_50_ values for inhibition of IMPα:NLS interactions.

IC_50_(μΜ) *				
Binding Interaction	Bay 11-7085	CAPE	Auranofin	MMV003270
PfIMPα:TGS1-NLS-GFP	1.9 ± 0.1	1.8 ± 0.3	0.07 ± 0.01	0.3 ± 0.2
TgIMPα:T-ag-NLS-GFP	30.4 ± 0.6	2.9 ± 0.9	0.08 ± 0.01	0.2 ± 0.1
MmΔIBBIMPα:T-ag-NLS	23.4 ± 2.9	0.7 ± 0.1	0.06 ± 0.01	0.4 ± 0.2

* Results represent the mean ± SEM (*n* = 3) for IC_50_ values measured as per Figure 3.

**Table 3:** Summary of the IC_50_ values for inhibition of *P. falciparum* and *T. gondii* parasites by IMPα inhibitors in culture.

Compound	IC_50_ (µM) *	
	*P. falciparum*	*T. gondii*
auranofin	1.9 + 0.5	2.3 ± 0.8
CAPE	7.5 ± 1.1	>10 ^#^
Bay 11-7085	>10 ^#^	2.7 ± 0.7
DHA	0.001 + 0.0005	NT ^x^
pyrimethamine	NT ^x^	0.46 ± 0.14 ^

* Results represent the mean ± SD (*n* = 3) for IC_50_ values determined as per Figure 6 and Figure 7. ^#^ See Figure 5. ^x^ NT, not tested. ^ See also [54].

## Data Availability

Not applicable.

## Supplementary Materials

The following are available online at https://www.mdpi.com/article/10.3390/cells11071201/s1, Figure S1: Low toxicity of auranofin and Bay 11-7085 in HFF cells. Freshly confluent HFF cells were treated with increasing concentrations of auranofin and Bay 11-7085 as indicated for 48 h followed by an MTT assay, performed as described in the Section 2. Results represent the mean ± SD for duplicate wells from a single assay, representative of 2 independent experiments (see Table S1 for pooled data). Table S1: Summary of IC50, CC_50_ and Selectivity Index data for auranofin and Bay 11-7085 in *T. gondii* tachyzoites/HFF host cells.
