# Supplementary material for: High-Throughput Screening to Identify Inhibitors of Plasmodium falciparum Importin α
Source: Cells. 2022 Apr 2;11(7):1201. doi: 10.3390/cells11071201 (PMC8997399; doi:10.3390/cells11071201)
Supplement: Supplementary file 1 [file cells-11-01201-s001.zip › cells-1646609-supplementary.pdf]

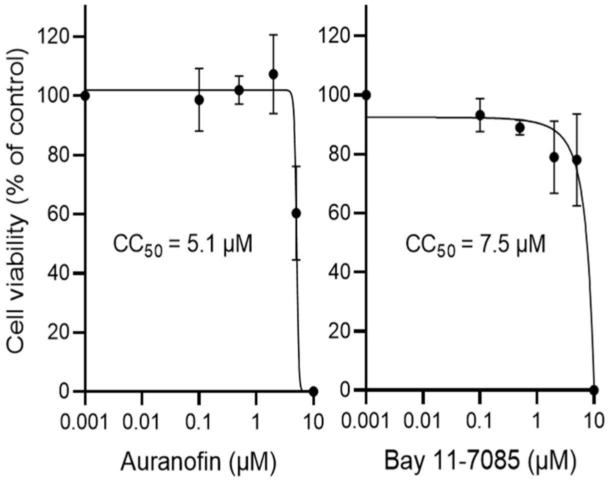

Figure S1. Low toxicity of auranofin and Bay 11-7085 in HFF cells. Freshly confluent HFF cells were treated with increasing concentrations of auranofin and Bay 11-7085 as indicated for 48 h followed by an MTT assay, performed as described in the Materials and Methods section. Results represent the mean ± SD for duplicate wells from a single assay, representative of 2 independent experiments (see Table S1 for pooled data).

Table S1: Summary of IC50, CC_{50} and Selectivity Index data for auranofin and Bay 11-7085 in *T. gondii* tachyzoites/HFF host cells.

Compound	IC_{50} (μM) *	CC50 (μM) **	S/I
Bay 11-7085	2.7 ± 1.2	8.2 ± 0.7	3
auranofin	2.3 + 1.4	5.1 ± 0.0	2.2
pyrimethamine	0.5 ± 0.1	>> 10 μM	22

* Results represent the mean ± SEM (*n* = 3) from experiments as per Figure 7; ** Results represent the mean ± SD (*n* = 2) from experiments as per Figure S1.
